# A bistable hysteretic switch in an activator–repressor regulated restriction–modification system

**DOI:** 10.1093/nar/gkt324

**Published:** 2013-04-27

**Authors:** Kristen Williams, Michael A. Savageau, Robert M. Blumenthal

**Affiliations:** ^1^Department of Medical Microbiology & Immunology, and Program in Bioinformatics, University of Toledo, Toledo, OH 43614, USA and ^2^Biomedical Engineering Department, and Microbiology Graduate Group, University of California, Davis, CA 95616, USA

## Abstract

Restriction–modification (RM) systems are extremely widespread among bacteria and archaea, and are often specified by mobile genetic elements. In type II RM systems, where the restriction endonuclease (REase) and protective DNA methyltransferase (MTase) are separate proteins, a major regulatory challenge is delaying expression of the REase relative to the MTase after RM genes enter a new host cell. Basic understanding of this regulation is available for few RM systems, and detailed understanding for none. The PvuII RM system is one of a large subset in which the central regulatory role is played by an activator–repressor protein (called C, for controller). REase expression depends upon activation by C, whereas expression of the MTase does not. Thus delay of REase expression depends on the rate of C-protein accumulation. This is a nonlinear process, as C also activates transcription of its own gene. Mathematical modeling of the PvuII system led to the unexpected predictions of responsiveness to a factor not previously studied in RM system control—gene copy number—and of a hysteretic response. In this study, those predictions have been confirmed experimentally. The results may apply to many other C-regulated RM systems, and help explain their ability to spread so widely.

## INTRODUCTION

Restriction–modification (RM) systems are extremely widespread among bacteria and archaea (1). They have been found in nearly every major bacterial group, save for some species that grow exclusively inside eukaryotic host cells. The physiological roles of most RM systems have not been tested, but many of them play roles in defense against infecting bacteriophages (2,3), and they may also promote gene exchange by generating recombinogenic ends on incoming DNA and by physically separating potentially advantageous from potentially deleterious genes (4).

The genes for RM systems are often specified by mobile genetic elements (5); for example the PvuII genes are plasmid borne (6,7). In Type IIP RM systems, where the restriction endonuclease (REase) and protective DNA methyltransferase (MTase) are separate proteins (8), a major regulatory challenge is delaying expression of the REase relative to the MTase after the RM genes enter a new host cell. The perils of misregulating Type IIP RM systems are illustrated by the fact that at least some of them behave as toxin–antitoxin systems, with the REase as toxin and the MTase as antitoxin, and exhibit ‘selfish’ behavior (9–11). That is, loss of the genes for the RM system can result in death of the host cell, as protective methylation decreases before REase activity disappears. Despite their importance, basic understanding of gene regulation is available for few RM systems, and detailed understanding for none.

Five regulatory strategies have been identified among RM systems (henceforth we refer only to Type IIP RM systems). These include methylation feedback, MTase autorepression, promoter competition, antisense RNAs and C proteins. The first two interfere with the MTase gene promoter via DNA methylation and/or by direct MTase binding (12–16). This helps prevent overexpression of MTase, which could compromise effectiveness of the REase; but these strategies, by themselves, do not explain how REase expression is delayed in new host cells. Promoter competition has been reported in the Ecl18kI system, where RNA polymerase binding to the MTase gene promoter prevents binding to the nearby REase gene promoter; in combination with MTase autorepression, accumulation of MTase relieves the promoter competition and allows REase expression (17). The antisense RNA strategy is best studied in the EcoRI and Eco29kI systems (18–20). For example, a small RNA complementary to the 3′ end of the REase gene decreases EcoRI REase expression, even when the RNA is supplied *in trans*. However, the contribution of this RNA to kinetics of EcoRI gene expression has not yet been determined.

**Figure 1.**
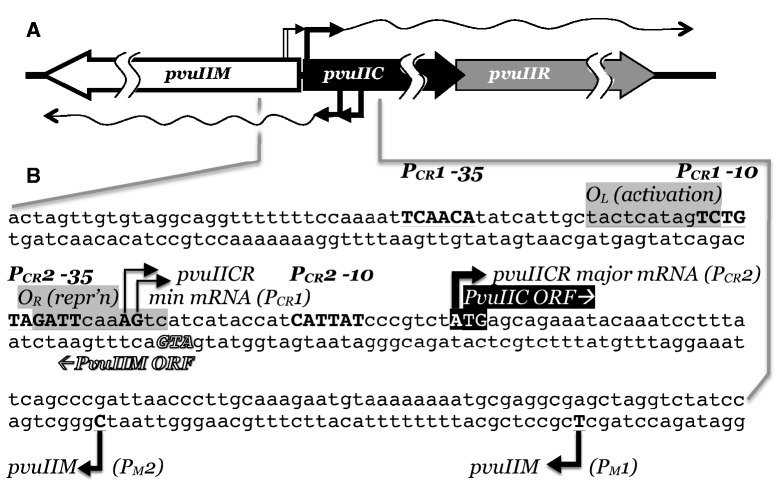
Transcriptional regulatory region of PvuII RM system. (**A**) Genetic map of PvuII system. The three genes *pvuIIM*, *pvuIIC* and *pvuIIR* code, respectively, for the MTase, activator–repressor and endonuclease. Bent arrows followed by wavy lines indicate promoters and their mRNAs. (**B**) Sequence of the regulatory region. The sites of C.PvuII activation/repression, the initiation codons for *pvuIIM* and *pvuIIC*, and the transcript 5′ ends, are from experimental data (see text for references); the locations of the −10 and −35 hexamers are inferred from the transcript starting points. P_CR_1 is a very weak promoter, repressed by C.PvuII, that is believed to initiate the autogenous activation cycle by providing a low initial amount of *pvuIIC* transcription; P_CR_2 is the much stronger promoter that is controlled by C.PvuII. P_M_1 and P_M_2 are responsible for transcription of *pvuIIM*.

The PvuII RM system is one of a substantial subset in which the central regulatory role is played by an activator–repressor protein (called C, for controller; Figure 1) (21–23). REase expression is dependent on activation by C, whereas expression of the MTase is not. Thus, in simple terms, delay of REase expression depends on the time it takes for sufficient C protein to accumulate. This delay of ∼10 min was measured in the case of PvuII, after the RM genes (moved to an M13 bacteriophage vector) were introduced over a short period of time into a growing population of cells (24). The C-dependent regulatory system is nonlinear, as C also activates transcription of its own gene. Positive feedback loops can be associated with extended induction times under certain conditions (25–27), which in mobile RM systems might be a desirable feature; though this effect would in any case be tempered in systems such as PvuII by the weak second promoter (independent of C activation) that provides a basal amount of the activator (28). The autogenous activation by C is a double-edged sword—it can result in desirable switch-like behavior, with a period of low C (and REase) levels followed by a rapid transition to substantial expression of both, but could also result in potentially dangerous overexpression of the REase. This may explain why, in at least several tested cases, C is not only an autogenous activator, but also an autogenous repressor. Indeed, when the repression site bound by C.PvuII was disrupted while its activation site was left intact, a huge increase in C-dependent transcription was seen (21).

Given the critical importance of maintaining an appropriate balance between MTase and REase activities, it is noteworthy that negative autogenous regulatory circuits have been shown, both theoretically and experimentally, to increase gene expression stability (29,30), whereas positive autogenous regulatory circuits can exhibit hysteretic behavior, where expression is influenced by the recent history of the cell (26,31). Regulation by C protein, which involves both positive and negative autogenous regulation, might benefit from a combination of advantages. Although hysteretic expression has not been demonstrated for RM systems, it theoretically would make their control more robust with respect to random perturbations. Specifically, cells that had recently expressed C protein (and thus REase) at low levels would have a higher induction threshold than cells that had recently expressed C and REase at higher levels, helping to prevent premature expression of REase.

The purpose of this study was to use a combination of mathematical modeling and laboratory experimentation, to assess the possibility that C-regulated RM systems such as PvuII exhibit hysteretic bistable regulatory behavior. We report both that the PvuII RM system is hysteretic, and that this behavior is dependent in part upon an element not previously examined in RM system regulation—gene copy number.

## MATERIALS AND METHODS

### Mathematical modeling

We constructed and analysed models using the Design Space Toolbox for MATLAB 1.0 (32). We simulated deterministic models with the MATLAB stiff solver, ode15s, and simulated stochastic models with an implementation of the Euler–Maruyama method (33) in MATLAB. All tests were performed using MATLAB 7.8 (R2009a).

### Bacterial strains, plasmids and growth conditions

We used two *Escherichia coli K-*12 strains. The genotypes of EPI300 (the CopyControl™ *s*train; Epicentre) and TOP10 (Invitrogen) are both F- *mcrA Δ(mrr-hsdRMS-mcrBC)* φ80*dlacZΔ*M15 *ΔlacX*74 *recA*1 *endA*1 *araD*139 *Δ(ara, leu)*7697 *galU galK* λ*- rpsL* (Str^R^) *nupG tonA*. φ80lacZΔM15 contains the entire *lac* operon (though with part of *lacZ* deleted), including lacI^q^. Compared to TOP10, strain EPI300 carries P*araBAD-trfA* in addition.

To make pKW177, the *araE* gene was PCR amplified from pJAT13*araE* (Addgene) and inserted at the KpnI and EcoRI sites of pBad24 (34). pUC19 (cut with SmaI and SbfI) was ligated with *araE-araC* from pBad24 (cut with NaeI and SbfI). The new clone was cut with ApaLI, and the segment containing P*lac-araEC* was ligated into ApaLI-cut pACYC177 yielding pKW177. To make pKW178, the P*araBAD-pvuIIC* segment was isolated from pIM1 (21) by digestion with ApaLI and AgeI, and inserted into pKW177 at the ApaLI and AgeI sites. pKW3.4 was prepared by cloning the full PvuIIRM system by digesting pPvuRM3.4 (6) with EcoRI and BamHI, and inserting into pCC1 (the CopyControlTM vector; Epicentre) (35) cut with the same two enzymes.

In the arabinose titrations (see next section), some experiments used *E. coli* TOP10 carrying pDK435 (36) [based on pKK232-8 (37) and carrying P*pvuIICR-lacZ*], together with either pIM1 (21) or pKW21 (this work; both are based on the vector pBAD24, and both carry P*araBAD-pvuIIC*; pKW21 carries, in addition *araE*). In other experiments, *E. coli* EPI300 was used carrying both pKW3.4 (this work) and pKW177 (this work).

Experiments were carried out at 37°C, with aeration, in defined rich medium (MOPS rich, TekNova) containing glucose. Media were supplemented as appropriate with arabinose or antibiotics.

### Arabinose titrations

Arabinose titrations were used for two distinct purposes. Some experiments used *E. coli* TOP10 with pDK435 (P*pvuIICR-lacZ*) and either pIM1 or pKW21 (P*araBAD-pvuIIC* ± *araE*). In these experiments, increasing arabinose leads to increasing amounts of C.PvuII protein. In other experiments, *E. coli* EPI300 was used carrying both pKW3.4 and pKW177. In these experiments, increasing arabinose leads to increasing copy numbers of pKW3.4. For the hysteresis experiments, 20 doublings were allowed in the new medium before sampling for RNA analysis. Log phase cells in one of two starter cultures were diluted 1:10^6^ into media with a range of arabinose concentrations. For additional details, see text.

### QRT-PCR

Culture samples were removed directly into tubes containing RNA Protect™ (Qiagen) and stored as cell pellets at −80°C until use. Sample volumes were corrected for culture OD_600nm_ to maintain similar cell numbers. Samples were immediately mixed with 2 ml of RNA Protect reagent (Qiagen), and total RNA was isolated using the RNeasy Mini kit (Qiagen). cDNAs were obtained by using random hexamer primers (Invitrogen) and ImProm-II Reverse Transcriptase (Promega), per manufacturer’s protocols. Primers (Integrated DNA Technologies) were designed with Primer3 software to obtain a common T_m_ and similar PCR product sizes. The primers used are listed in Supplementary Table S1. A LightCycler (Roche) was used with manufacturer’s software. The PCR was performed in triplicate. Each reaction contained dNTPs (0.2 mM), PCR buffer (1x), primers (1 M), SYBR Green I dye (1:20 000) and Platinum Taq DNA polymerase (0.5 U; Invitrogen). The cycling parameters were 95°C for 2 min, followed by 40 cycles of 94°C for 5 s, 59°C for 5 s, 72°C for 15 s; and finally the melting curve (59°C–94°C) program for quality control. The mRNA levels for the target genes were quantified from the Ct value.

### Western blots

Culture samples were centrifuged at 16 000*g* for 5 min. The cell pellets were stored at −80°C until analysis. Pellets were resuspended in 2× SDS buffer (Invitrogen), and lysed by heating to 95°C for 10 min. Extracts were resolved on SDS-polyacrylamide gels (10%–20% Tris-Glycine NuPAGE Novex gradient gels; Invitrogen), and electroblotted to PVDF membranes. The blots were probed with a polyclonal rabbit antiserum against C.PvuII (21), and then with goat anti-rabbit antiserum conjugated to horseradish peroxidase (Strategic BioSolutions). Loading variation was determined by reprobing for the naturally biotinylated protein BCCP (38) with streptavidin conjugated to horseradish peroxidase (Sigma-Aldrich). In both cases, the peroxidase was detected with Super Signal West Pico chemiluminescent substrate (Pierce), per the manufacturer’s protocol, and luminescence measured in an Omega Molecular Imaging System (UltraLum).

## RESULTS

### Modeling the PvuII RM system

The three genes of the PvuII RM system are represented in Figure 1. Two oppositely oriented transcriptional units yield, respectively, the MTase (gene *pvuIIM*) and both the C protein and REase (*pvuIICR*). Each transcriptional unit includes two promoters (28,36). In the case of *pvuIICR*, the upstream promoter is weak but active in the absence of the PvuII C protein (C.PvuII), and is believed to initiate the positive feedback loop when the PvuII genes enter a new host cell. The downstream promoter (P*pvuIICR*2, or P_CR_2) is strongly activated, and also repressed, by C.PvuII. In a previous study, where P_CR_2 was fused to the *lacZ* reporter gene, steady-state exposure to an increasing range of C.PvuII levels led to a rise, then a sharp drop, in transcription (21). C.PvuII dimers bind cooperatively to two sites on the DNA—O_L_ binding is associated with activation and O_R_ with repression. In PvuII, O_R_ overlaps the P_CR_2 -35 hexamer (Figure 1), and selective disruption of O_R_ leads to greatly increased *lacZ* transcription in the model system just described (21). In studies of a different C-dependent RM system (AhdI), there is evidence for competition between RNA polymerase and C protein for the O_R_ site (39).

In attempting to understand the C-dependent regulation of RM systems, we used two types of mathematical modeling. In one, modeling was focused specifically on the PvuII system; the parameter values were estimated and the behavior simulated (not shown). The goal of this type of modeling is to see if the model faithfully reproduces known behavior of the real system, and predicts real behavior that was previously unknown. The AhdI system has been usefully modeled in this way (39), and includes some features relatively unique to that system, such as methylation feedback (see Introduction). In the second type, modeling was applied to the large C-regulated class of RM system (22,23) that includes PvuII, looking for qualitative behavior that reflects system architecture and the signs (+ or −) of the interactions. For this type of modeling, quantitative values are not required for all the parameters; indeed, this approach is most useful for teasing out design principles of systems for which values for several parameters are not yet available. The goal is to obtain qualitative predictions that apply to an entire class of systems (26,30,32,40–43).

**Figure 2.**
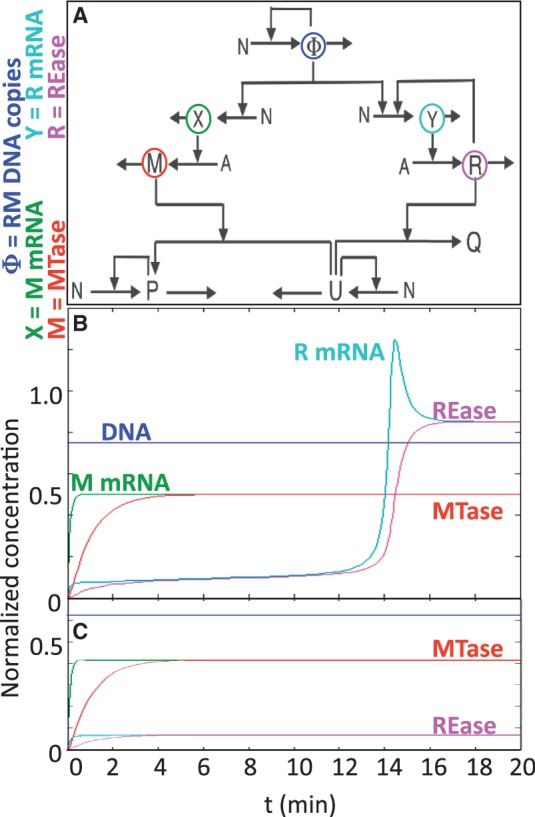
Modeling C-dependent RM systems. (**A**) Core circuit, generalized for C-dependent systems. In this initial model, C and R are combined (as R) since, in virtually all known C-dependent systems, they are cotranscribed. Chromosomal DNA is represented as P (Protectively methylated) U (Unprotected) and Q (cleaved). Other variables are defined on the left of the figure. *N* represents nucleotide pools (NTPs and dNTPs, as appropriate), whereas *A* represents aminoacyl-tRNA pools. (**B**, **C**) Simulations were carried out at two different fixed values of phi (gene copy number): *B* = 11 copies, *C* = 8 copies.

A schematic diagram of the mathematical model is shown in Figure 2A. This version of the model focuses on the RM system in isolation, ignoring for now issues such as the competition of changing levels of bulk DNA for the activating C protein, or changes in the levels of available RNA polymerase or ribosomes. For completeness, and because the PvuII genes naturally reside on a plasmid, gene copy number was included in the model (Φ), though population noise in copy number was not (44). The native plasmid, pPvu1, includes *mob* genes (7), implying that it could transfer between cells via conjugation (in the presence of a conjugative plasmid) (45). This would result in the genes entering in single copy, followed by a rise to the normal copy number of the plasmid (which may depend on environmental conditions). Thus it seemed prudent to include gene copy number in the model.

Simulations of PvuII gene expression kinetics were run at different fixed gene copy numbers and, unexpectedly, predicted a sharp threshold effect. Below a given copy number (Figure 2C), MTase levels rose to a stable pleateau but REase levels indefinitely remained very low. In contrast, above the copy number threshold, MTase levels behaved essentially as at low copy number, but REase levels spiked after a 12–14 min delay and plateaued at a substantially higher level. The model-predicted threshold was 11 gene copies, but given the number of estimated parameters we were most interested in whether or not a sharp threshold really existed at all.

We proceeded to test the model’s predictions in two ways, based on the two physiological ramps likely to be encountered by an RM system such as PvuII after it enters a new host cell: increasing levels of the C activator–repressor, and increasing DNA copy number. Accordingly, we first determined the kinetics of responses to varied amounts of C.PvuII, at fixed gene copy number. Next, we adapted a method for varying the steady-state copy number of a plasmid, and determined the effects of copy number and previous culture conditions on PvuII gene expression.

### Kinetics of response to C.PvuII

**Figure 3.**
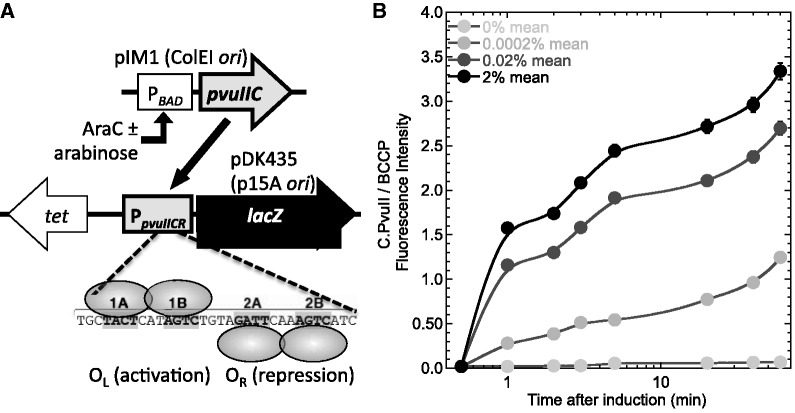
Induction kinetics of *pvuIICR* operon. (**A**) System used. To allow stable titration with C.PvuII, its positive feedback loop was broken by placing the *pvuIIC* gene under control of the arabinose-inducible P*araBAD* promoter (top), while fusing the *lacZ* reporter gene (β-galactosidase) to the promoter that normally controls *pvuIIC* and is regulated by C.PvuII (P*pvuIICR*, middle; including both P_CR_1 and P_CR_2). The strain background was *E. coli* TOP10. The *tet* gene (tetracycline resistance) is on the same plasmid as *lacZ*, and was used to normalize gene expression in some experiments. The C.PvuII binding sites are shown at the bottom, with ovals representing C.PvuII homodimers. Some experiments use a non-repressing variant, in which C-box 2B (bottom) is altered (AGTC → GATC). For references, see text. (**B**) C.PvuII levels. Results of C.PvuII measurements from western blots of cell extracts. C.PvuII was detected by a polyclonal primary antiserum, with final readout via luminescence densitometry. Means of triplicates, ±SE, are shown. The loading normalization was to BCCP, a naturally biotinylated *E. coli* protein. See Methods for details.

The first perturbation we used to test the model was a range of increases in concentration of the activator–repressor, C.PvuII. A steady-state *in vivo* titration analysis was previously carried out (21), but not a kinetic analysis. As with that steady-state titration, we used a two-plasmid system (Figure 3A) with the gene for C.PvuII fused to an arabinose-inducible promoter (P*araBAD-pvuIIC*), and the promoter controlled by C.PvuII fused to a reporter gene (P*pvuIICR-lacZ*). The different arabinose levels had no significant effect on growth rates (Supplementary Figure S1).

We first characterized the arabinose-dependent induction times of *pvuIIC* mRNA and C.PvuII protein in this system, to provide a reference point for examining response of the P*pvuIICR* promoter. C.PvuII is an 84 aa polypeptide, which should take 6 s to be translated, at the elongation rate of 14 aa/s (46). Supplementary Figure S2 (panel A) shows the transcriptional response, with induced levels diverging from the baseline 1–4 min after arabinose addition, and continuing to rise for at least 90 min. However for the purposes of this study, the more relevant parameter is levels of the C.PvuII protein. A western blot quantitation is shown in Figure 3B. The blots were probed with a polyclonal antiserum against C.PvuII, and normalized for loading variation by reprobing for the naturally biotinylated *E. coli* protein BCCP. Induction is clear within 1 min, and is still rising after 1 h. In this system, background production of C.PvuII protein was undetectable. The protein/mRNA ratios for *pvuIIC* are not proportional (Supplementary Figure S2, panel B) and, while they may suggest an interesting translational regulatory phenomenon, they clearly indicate the importance of using protein rather than mRNA measurements for interpreting the following experiments.

**Figure 4.**
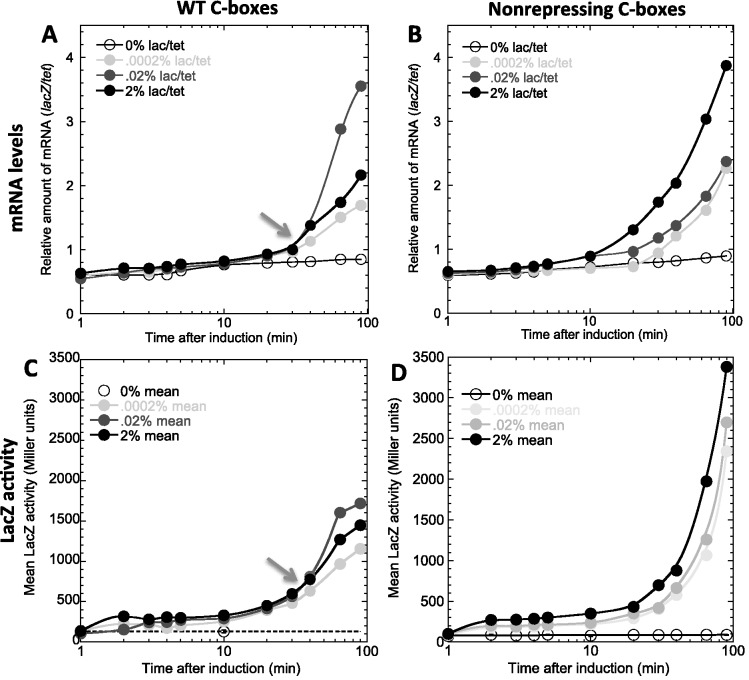
Temporal response of P*pvuIICR* to inductions of PvuIIC. Top (**A**, **B**) shows *lacZ* mRNA levels measured by QRT-PCR; bottom (**C**, **D**) shows β-galactosidase activities. Left (A, C) is with the WT P*pvuIICR*2 promoter; right (B, D) is the non-repressing variant altered in O_R_. The arrows indicate crossover points, at which a higher inducer level results in lower responses. Such crossovers are absent in titrations using the non-repressing mutant.

We next assessed activity of the WT P*pvuIICR* promoter in response to these temporal ramps of C.PvuII, by measuring expression of the linked *lacZ* gene (Figure 4). The *tet* gene, on the same plasmid as the *lacZ* gene, served as the reference mRNA. At various times after adding inducer, portions of the culture were assessed for β-galactosidase activity or, after being fixed (to stop transcription and stabilize existing mRNA), *lacZ* mRNA was quantitated via QRT-PCR. In panel A, there is a consistent ∼10 min delay between arabinose addition and appearance of *lacZ* mRNA. Some of this lag reflects the accumulation of C.PvuII (included in the model), some is the searching time for C-boxes amid the huge excess of non-C-box DNA (not included in the model), and some is the time it takes for RNA polymerase to elongate from P*pvuIICR* to the region of *lacZ* used for QRT-PCR (roughly 70 s at ∼40 nt/s (46,47); included in the model for *pvuIICR*, but *lacZ* is much larger than *pvuIICR*).

Strikingly, about 30 min after arabinose addition, as indicated by the arrow (Figure 4A), the highest arabinose (and C.PvuII) level began to yield *less* transcription from P*pvuIICR* than did a lower amount of arabinose. The existence of a crossover point was predicted from the known dual function of C.PvuII as both activator and repressor (21), but to test this interpretation we repeated the experiment with the non-repressing variant of P*pvuIICR* (Figure 4B). Unlike the WT results, the non-repressing variants show no sign of approaching the maximal expression rate within the 90 min period of the experiment. More importantly, there is no crossover and the induction responses increase monotonically with arabinose concentration.

The β-galactosidase activity (Figure 4, panels C and D) reveals a comparable pattern. Specifically there is a crossover point, about 30 min after *pvuIIC* induction, with the WT promoter (arrow in panel C) but not with the non-repressing variant. For reasons described in the following section, we tested whether constitutive expression of the arabinose transporter *araE* would affect these results, but found it had only marginal effects (Supplementary Figure S3; perhaps due to the limited timecourse used).

### Effects of gene copy number

**Figure 5.**
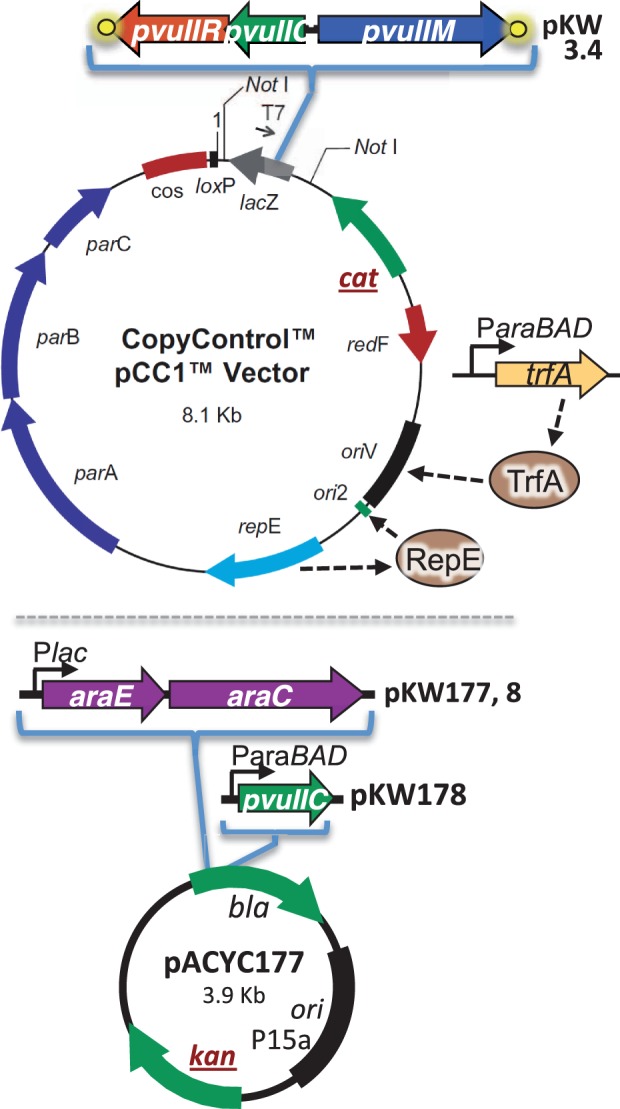
Plasmids used for copy number variation experiments. pKW3.4 (upper) is the pCC1 vector (Epicentre Technologies, Madison, WI), with the PvuII RM system inserted. As described in the text, some variants of this plasmid lack the Avi tags on *pvuIIR* and/or *pvuIIM* (yellow circles). In standard *E. coli* host strains, replication of this plasmid is controlled by RepE acting at *ori*2 and results in very low copy number. In the Epicentre CopyControl™ *E. coli* host EPI300, copy numbers can be induced to higher levels via a chromosomally coded *trfA* gene. The responsiveness of this system to induction was improved by adding a second plasmid, pKW177 (lower), which is the low copy vector pACYC177 with two inserted genes. One is *araC*, which specifies the arabinose-responsive regulator and provides a lower background (uninduced) copy number for pCC1. The second gene is *araE*, the arabinose transport protein, which eliminates the population inhomogeneity that results at intermediate inducer levels, due to a positive feedback loop involving induction of inducer uptake. pKW178 has, in addition to the genes in pKW177, the *pvuIIC* gene under control of P_BAD_. In strains other than the CopyControl™ *E. coli* strain EPI300, arabinose does not affect pKW3.4 but induces expression of *pvuIIC* from pKW178 if that plasmid is present. The genes *cat* (chloramphenicol resistance) and *kan* (kanamycin resistance) are used as indicators of copy number for the two plasmids in subsequent experiments. See the text for references.

We next tested the effects, on PvuII gene expression, of systematically varying the copy number of the PvuII genes. To do this, we adapted a system originally developed for the purpose of having a plasmid that could yield very low copy numbers for experiments, while allowing amplification to high numbers to facilitate purification and modification (35). This system is based on a plasmid, pCC1, that has two separate replication origins (Figure 5, upper). One, *ori2* derived from the plasmid F, responds to the pCC1-coded RepE protein and results in maintenance of low copy number (1-2 per chromosome equivalent of DNA). The other, *oriV* from the IncP plasmid ColEI, responds to the protein TrfA and generates high copy numbers (in the absence of genes that normally limit copy number). However *trfA* is not on pCC1, but in the EPI300 chromosome, fused to the arabinose-inducible P*araBAD* promoter. [Note that in some other experiments in this study, P*araBAD* and arabinose are used to control expression of *pvuIIC*, in a host strain lacking *trfA*. In this section and the next, arabinose controls plasmid copy number and has no *direct* effect on *pvuIIC* expression.]

**Figure 6.**
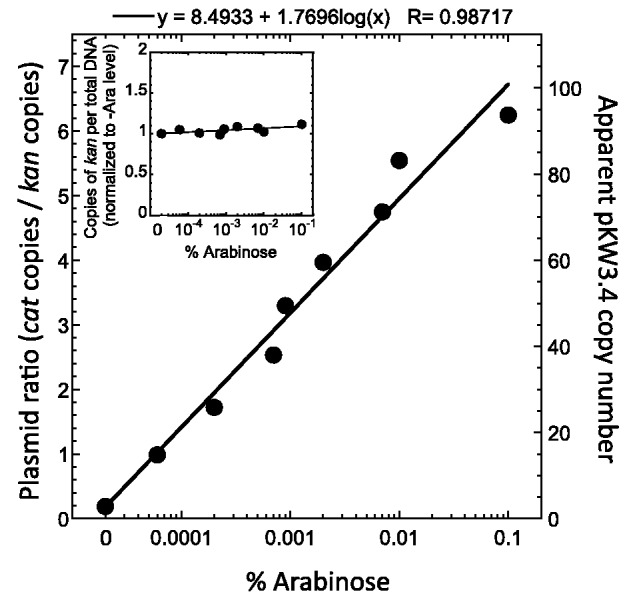
Induction range of plasmid copy number. *E. coli* strain EPI300, containing the two plasmids pKW177 and pKW3.4 (Figure 5), was grown overnight in MOPS-rich medium containing various amounts of arabinose, using inocula such that the cultures never reached stationary phase. These were used to inoculate cultures at the same arabinose levels. Plasmid DNA was extracted in mid-logarithmic phase, and the *cat* (pKW3.4) to *kan* (pKW177) copy number ratios determined via QRT-PCR. The inset shows *kan* copies per fixed amount of total cell mass. The apparent copy number of pKW3.4 (right axis) is determined by multiplying the plasmid ratio by the measured copy number of ∼15 pACYC184 per chromosome equivalent of DNA (see text for references).

We found it necessary to make two modifications to this system in order to achieve a smooth and predictable variation of copy number with inducer concentration. First, when maintained in the *E. coli* strain EPI300, we found the background (uninduced) pCC1 copy number was substantially higher than when in strains lacking the *trfA* gene. This suggested significant background expression of *trfA*, at least under our growth conditions (MOPS-rich medium including glucose; see Methods). We thus introduced a compatible second plasmid carrying the *araC* activator–repressor gene (Figure 5, lower; plasmid pKW177 does not carry *pvuIIC*). In the absence of arabinose, this led to increased repression of P*araBAD-trfA*, as evidenced by a lower baseline pCC1 copy number (not shown). In addition, *E. coli* EPI300 has a functional arabinose transporter, specified by *araE*, that is itself induced by arabinose. Induction of the transporter greatly sensitizes the cell to exogenous arabinose, and at intermediate levels of the inducer the cells can be in two distinct subpopulations showing very different levels of induction (48). To avoid this, our second modification was placing *araE* on the second plasmid, similar to an approach taken earlier (49). As illustrated in Figure 5, these two *ara* genes are under control of the IPTG-inducible P*lac*, though in practice the background levels of expression were sufficient to allow stable, smoothly increasing pCC1 copy number with increasing concentrations of arabinose (Figure 6). pKW3.4 carries the PvuII genes in the pCC1 vector (Figure 5, upper).

Unlike the experiments in the previous section (C.PvuII pulse), these experiments employed cultures growing in an approximation of steady state. Separate starter cultures were grown for each tested concentration of arabinose, and diluted in mid-log phase into the larger culture of the same medium. As reference genes for these studies, we used the antibiotic resistance genes on the two plasmids. The *kan* gene on the pACYC177-derived *araEC* plasmid should not change copy number in response to arabinose. The inset in Figure 6 shows no arabinose-dependent variation in the ratio of *kan* to total DNA. In contrast, the *cat* gene on the pCC1-derived plasmid carrying the PvuII genes should increase in copy number with increasing arabinose. The main part of Figure 6 shows a predictable relationship between arabinose levels and copy number, ranging from ∼2 to ∼95, based on a copy number of 15 for the pACYC177-based *kan* plasmid (50). For comparison, an antibiotic-resistant derivative of the original PvuII plasmid pPvu1 (from *Proteus vulgaris*) yielded a copy number of 13-15 (in *E. coli* grown in rich medium) (6,7).

**Figure 7.**
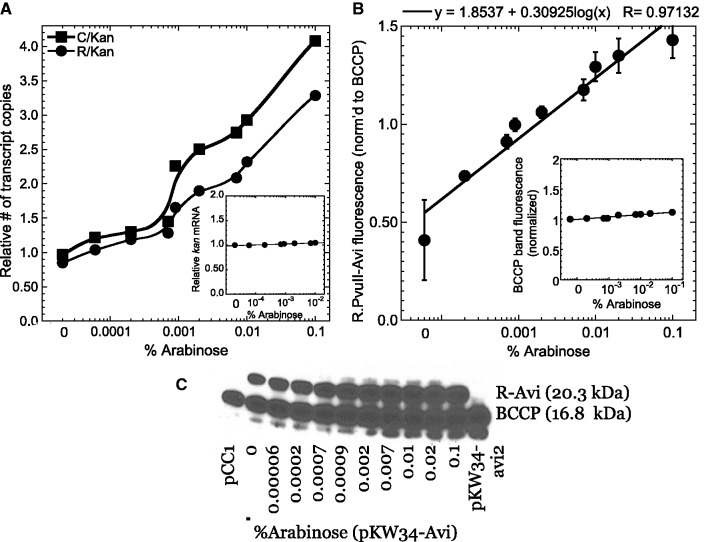
PvuII gene expression in response to gene copy number. (**A**) Transcriptional responses. Reverse transcript QRT-PCR was used to determine the levels of the mRNAs for the Control protein (C, squares), and REase (R, circles) relative to that for *kan* (inset, normalized to total RNA). The experiment was carried out as described in Figure 6. (**B**, **C**) Translational responses. Panel (B) shows densitometry of triplicate western blots (mean ± SE) such as the one shown in panel (C). Note that panel (C) is taken from a film image exposed to maximize visibility, while the quantitation is from luminescence densitometry. The PvuII REase (R) includes a carboxyl-terminal Avi tag (55), that is biotinylated *in vivo*. Levels were normalized to BCCP, the one *E. coli* K-12 protein that is naturally biotinylated (inset in panel (B), relative to constant total protein). Panel (C), probed with streptavidin-HRP, indicates the positions of size markers (left), and two control extracts from the vector pCC1 (only BCCP is labeled) and pKW34-avi2 [only BCCP and MTase (latter not included in section of blot shown) are labeled].

The effects on PvuII gene expression are shown in Figure 7. Panel A shows cDNA (mRNA) copies, normalized to those from the fixed-copy *kan* gene; the inset shows *kan* cDNA copies normalized to total cDNA, and reveals no significant variation with arabinose level. Since the intact, WT PvuII RM system genes are on the pCC1 plasmid, the default assumption would be that all gene products scale monotonically with copy number. At the translational level, this appears to be a valid assumption (Figure 7, panels B and C). The REase gene carries a carboxyl-terminal Avi tag, that results in biotinylation *in vivo* (51) (again, naturally biotinylated BCCP protein served as an internal loading reference).

However the transcriptional responses are a more direct measure of P*pvuIICR* activity, and both *pvuIIC* and *pvuIIR* mRNA levels show a discontinuity with respect to plasmid copy number (Figure 7A). The transition begins at ∼0.0008% arabinose, corresponding (per Figure 6) to a copy number of ∼45. While the transition point is at a higher copy number than predicted by the initial model, presumably due to our need to use estimates for some of the parameter values, this result is consistent with a key prediction of the model. Accordingly, we examined this further by testing for hysteretic behavior.

### Hysteresis in PvuII gene expression

Hysteresis in gene expression refers to responsiveness not only to the current level of a given regulatory signal, but also to the recent history of that signal’s levels (26,31). The combination of positive and negative feedback loops in C-dependent RM systems such as PvuII is often associated with hysteretic behavior (52,53), and the discontinuous response of P*pvuIICR* to copy number (Figure 7A) was consistent with switching and possibly bistability.

**Figure 8.**
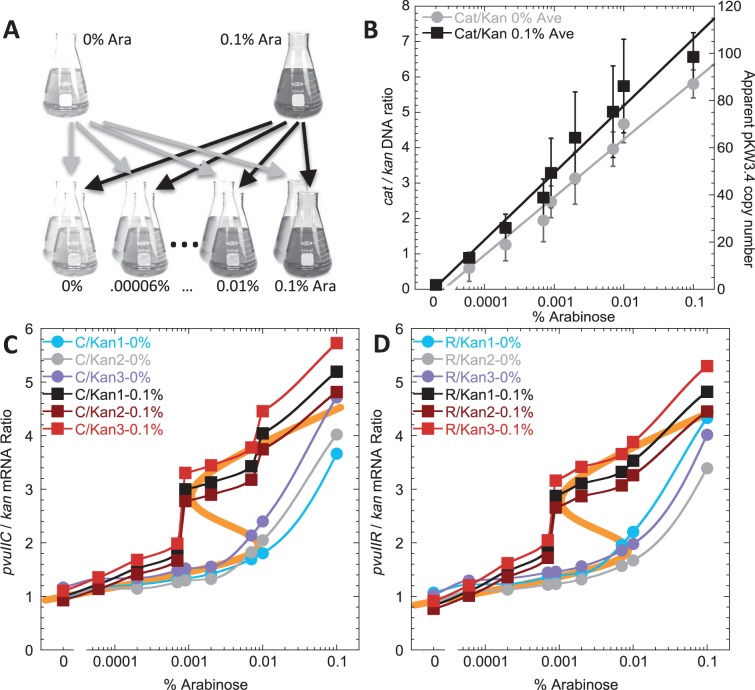
Hysteresis in the PvuII RM system. (**A**) Summary of the experiment. Two overnight cultures (that did not reach stationary phase) were grown in MOPS-rich medium containing either 0% or 0.1% arabinose. These were then used to inoculate medium containing a range of arabinose concentrations. This experiment was done in triplicate, starting with three different colonies. RNA was then extracted and subjected to reverse transcription QRT-PCR, using *kan* mRNA levels as the baseline. (**B**) Relative plasmid DNA copy number. The experiment was carried out as in (A), but samples analysed as in (Figure 6) to determine copy number of the pCC1-based plasmid. Symbols indicate the averages from two experiments, and bars indicate the range. Gray circles indicate cultures diluted from the starter culture lacking arabinose; black squares indicate cultures diluted from the 0.1% arabinose starter culture. (**C**) Results for the C-protein gene, *pvuIIC*. The three culture sets inoculated from the 0% arabinose overnights are shown as circles, and the three started from 0.1% overnights shown as squares. The thick orange line in the background shows the results of a mathematical model fit to the data. (**D**) Results for the REase gene, *pvuIIR*, otherwise as in (C).

To test this for PvuII, we repeated the copy number experiments from the previous section with one change. Instead of growing multiple starter cultures in the various arabinose concentrations at which that culture would later be grown for the experiment, we used two different log phase starter cultures (0% and 0.1% arabinose) and used each of them to inoculate a series of triplicate cultures at varying arabinose levels (Figure 8A). Cultures were diluted 1:10^6^ and grown for ∼20 doublings in the new medium (54), to mid-log phase, before fixation and RNA extraction.

The most important result is that the two starter culture conditions led to markedly different results, for both *pvuIIC* and *pvuIIR* mRNA (Figure 8, panels C and D). The cultures originating from +Ara starters (squares) showed a sharp threshold at ∼0.0008%, whereas those from the –Ara starters (circles) exhibited a much more gradual increase. As predicted, and as an internal control, no evidence of hysteresis was seen for the MTase gene *pvuIIM* (unpublished observation). Furthermore, this hysteresis was not apparent in the plasmid copy numbers (Figure 8B).

A model giving the best fit to the data is shown (orange line in panels C and D of Figure 8). As noted earlier, in the initial formulation of the model (Figure 2A), no distinction is made between C.PvuII mRNA and R.PvuII (REase) mRNA, as they are cotranscribed and no independent *pvuIIR* promoter is evident (28). The change in *pvuIICR* bicistronic mRNA, Y, is given by the difference in the rate of synthesis and the rate of loss with first-order rate constant 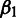. The rate of synthesis is modeled as a rational function that has a minimum or basal value 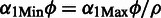, where 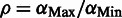, in the absence of the C-protein activator–repressor (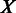). Y increases in a sigmoid fashion toward 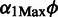 as 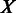 exceeds the concentration for half-maximal activation, 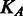. With further increases in C protein, the rate begins to decrease toward 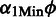 as 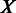 exceeds the concentration for half-maximal repression, 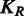. The cooperativity associated with the activation is given by the Hill coefficient (*p*), and that associated with the repression by *n*.

The change in C.PvuII (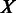) is given by the difference in the rate of synthesis, 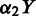 that is proportional to the concentration of the *pvuIICR* mRNA, and the rate of loss with first-order rate constant 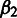.

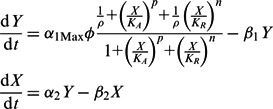



In steady state, these two equations can be combined to yield the following algebraic equation that relates copy number to C.PvuII levels:

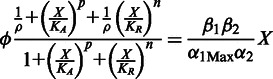


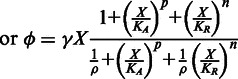



This is the equation used to fit the experimental data. The values for the parameters are the following: 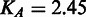, 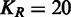, 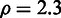, 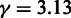, 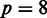 and 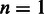. The relationship between 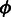 and %Ara used in the fitting was 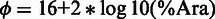. The parameter 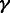 simply shifts the entire curve along the 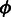 (%Ara) axis without changing the shape. The capacity for regulation, 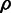, roughly determines the difference in the slopes at the extreme values of %Ara.

The model (S-shaped orange curve) predicts three possible steady states in the middle of the copy number range: two represent stable steady states, and those are the ones that fit the experimental data. The third steady state (middle of S-curve, with no data points) is unstable, exists only transiently, and would not generally be observed experimentally. The most influential parameters in providing a good fit to the data in Figure 8 are the cooperativity for activation, 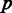, and the half-maximal concentration for activation, 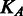. In contrast, 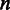 and 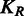 (the repression terms) are less critical. Our data suggest that one of the hysteretic thresholds occurs at a copy number of ∼10. The best fit to the data yields an estimate of 3.1 for the ratio of the rate constant for C loss (β_1_) to the maximum rate of C synthesis per gene copy (α_1Max_). For cells growing exponentially with a doubling time of 1 h and a completely stable C protein, the first-order rate constant for C removal is the dilution rate (0.693/h) and V_max_ is 0.22, where concentration units are hour/gene copy. A faster doubling time or a more labile C protein would shift the entire hysteretic pattern to higher copy numbers. Conversely, slower growth would shift the hysteretic pattern to lower copy numbers. Such shifts could also be caused (in a reciprocal fashion) by changes in the maximal rate of C gene transcription.

## DISCUSSION

RM systems play important modulatory roles in the flow of genetic information between bacterial and archaeal cells. Their relative ubiquity among these cells suggests their importance, and their frequent presence on mobile genetic elements indicates that RM genes routinely move into new host cells. Thus a key feature of RM system gene control is ensuring a lag, presumably as short as possible, between the appearance of MTase and REase activities. This lag is required to protectively methylate the new host cell’s DNA, and prevent REase-dependent lethal damage to the chromosome, as illustrated by the inability to transform cells with the PvuII RM system genes if they already contain the *pvuIIC* gene (11,28). Determining the basis for such regulation will deepen our understanding of the roles of RM systems, including their effects on gene flow among bacteria and archaea.

Guided by our modeling analysis, we explored two temporal phenomena to which the production of PvuII REase was predicted to be responsive: biosynthesis of the activator–repressor C.PvuII, and copy number of the PvuII genes. In both cases, behaviors predicted by the model were found experimentally, though the results suggested that some of the parameter estimates need to be revised.

With respect to growth in the absence of C.PvuII, followed by sudden initiation of its synthesis, transcription from the C.PvuII-controlled P*pvuIICR*2 promoter rose after a lag of ∼10 min. A portion of this lag is systemic, involving the time for C.PvuII to find the target promoter among the excess of other DNA in the cell, and the time for the long *lacZ* gene to be transcribed (though at 42 nt/s (46), that would take <2 min). The C.PvuII searching time may be increased in this model system, where *pvuIIC* is expressed on a plasmid separate from the one carrying P*pvuIICR*—in the native system, proximity of C.PvuII translation to the target promoter may greatly reduce searching times. The extent of the lag did not depend on the level of induction of *pvuIIC*, which varies substantially with different arabinose concentrations at post-induction times less than 10 min. For example, the amount of C.PvuII protein that accumulated in 0.0002% arabinose after 60 min was achieved within 1 min in 2% arabinose (Figure 3B); yet the responses from P*pvuIICR-lacZ* have the equivalent 10 min lag at both arabinose levels (Figure 4A). Significantly, this lag appears to be shorter in the variant of P*pvuIICR*2 in which the repression site has been selectively mutated (Figure 4B).

C-protein-associated promoter regions typically have two apparent binding sites for the C-protein homodimers (21,22,56). There is high binding cooperativity between O_L_ (activation) and O_R_ (repression) sites, for at least C.PvuII and C.AhdI (21,57). So it is noteworthy that even at the highest induction level of C.PvuII tested, repression never prevented activation of P*pvuIICR* though it did dampen the extent of the increase in transcription (Figure 4A). This may provide indirect support for the model in which RNA polymerase can occupy the promoter some fraction of the time, and occlude O_R_, while not initiating transcription until C protein occupies O_L_ (39).

To study the effects of gene copy number on expression of a C-protein-dependent RM system, we adapted the pCC1 system (35) as described in Results. We were able to obtain predictable copy numbers in proportion to the amount of inducer added (Figure 7). The results of copy number titration on transcription of the *pvuIICR* operon revealed a discontinuous response when approximating steady-state growth in a given concentration of arabinose (Figure 7A), and even more marked evidence for hysteretic bistability when cultures were shifted from high or low levels of inducer to a range of inducer concentrations and grown through ∼20 doublings before analysis (Figure 8). The discontinuity *vs.* copy number is not simply titration of a fixed amount of C.PvuII regulator, as the *pvuIIC* gene in these experiments is carried by the plasmid whose copy number is being varied (Figure 5, upper). To fit the data (orange lines in panels C and D of Figure 8), the model required a high cooperativity value for C.PvuII-dependent activation. This could reflect both homodimerization of the C.PvuII monomers as well as interaction with the two half-sites of O_L_ (Figure 1).

Importantly, the sensitivity to DNA copy number is not relevant only to plasmid-borne RM systems. Even for chromosomal RM genes, growth-rate-associated changes in the replication origin-to-terminus (*ori/ter*) ratio can differentially affect their dosage, as more-rapid growth is associated with relative amplification of origin-proximal genes (58–60). In *E. coli* the origin-to-terminus ratio can vary by over 8-fold. The onset of stationary phase would be expected to affect copy numbers for plasmid based [e.g., (61)] as well as chromosomal genes. According to our model and results, reduced copy numbers would tend to bias RM expression in favor of protective methylation. This difference would be accentuated by the apparent transcriptional attenuation between *pvuIIC* and *pvuIIR*, such that there is about twice as much *pvuIIC* mRNA as *pvuIIR* mRNA in growing cells (24).

Hysteretic expression has not previously been demontrated for RM systems, but could be valuable in lengthening the period of low REase expression immediately after the RM genes enter a new cell, while also expanding the conditions under which REase expression is elevated after the RM system has become established in the cell. Our results presumably apply broadly to the fairly large set of C-protein-controlled Type IIP RM systems. The possibilities of gene copy number responsiveness and hysteresis in other Type IIP RM systems that lack C proteins, and in other Type II systems (such as Type IIG, where the REase and MTase are fused), remain to be determined, though the same potential benefits would apply.

## SUPPLEMENTARY DATA

Supplementary Data are available at NAR Online: Supplementary Table 1 and Supplementary Figures 1–3.

## FUNDING

U.S. National Science Foundation [MCB0964728 to R.B.]; U.S. National Institutes of Health [GM030054-27 to M.S.]. Funding for open access charge: U.S. National Science Foundation.

*Conflict of interest statement.* None declared.

## Supplementary Material

Supplementary Data

## References

[1] Roberts RJ, Vincze T, Posfai J, Macelis D (2010). REBASE–a database for DNA restriction and modification: enzymes, genes and genomes. Nucleic Acids Res..

[2] Hoskisson PA, Smith MC (2007). Hypervariation and phase variation in the bacteriophage ‘resistome'. Curr. Opin. Microbiol..

[3] Labrie SJ, Samson JE, Moineau S (2010). Bacteriophage resistance mechanisms. Nat. Rev. Microbiol..

[4] McKane M, Milkman R (1995). Transduction, restriction and recombination patterns in Escherichia coli. Genetics.

[5] Wilson GG (1991). Organization of restriction-modification systems. Nucleic Acids Res..

[6] Blumenthal RM, Gregory SA, Cooperider JS (1985). Cloning of a restriction-modification system from Proteus vulgaris and its use in analyzing a methylase-sensitive phenotype in Escherichia coli. J. Bacteriol..

[7] Calvin Koons MD, Blumenthal RM (1995). Characterization of pPvu1, the autonomous plasmid from Proteus vulgaris that carries the genes of the PvuII restriction-modification system. Gene.

[8] Roberts RJ, Belfort M, Bestor T, Bhagwat AS, Bickle TA, Bitinaite J, Blumenthal RM, Degtyarev S, Dryden DT, Dybvig K (2003). A nomenclature for restriction enzymes, DNA methyltransferases, homing endonucleases and their genes. Nucleic Acids Res..

[9] Kobayashi I (2001). Behavior of restriction-modification systems as selfish mobile elements and their impact on genome evolution. Nucleic Acids Res..

[10] Naito T, Kusano K, Kobayashi I (1995). Selfish behavior of restriction-modification systems. Science.

[11] Nakayama Y, Kobayashi I (1998). Restriction-modification gene complexes as selfish gene entities: roles of a regulatory system in their establishment, maintenance, and apoptotic mutual exclusion. Proc. Natl Acad. Sci. USA.

[12] Beletskaya IV, Zakharova MV, Shlyapnikov MG, Semenova LM, Solonin AS (2000). DNA methylation at the CfrBI site is involved in expression control in the CfrBI restriction-modification system. Nucleic Acids Res..

[13] Christensen LL, Josephsen J (2004). The methyltransferase from the LlaDII restriction-modification system influences the level of expression of its own gene. J. Bacteriol..

[14] Karyagina A, Shilov I, Tashlitskii V, Khodoun M, Vasil'ev S, Lau PC, Nikolskaya I (1997). Specific binding of sso II DNA methyltransferase to its promoter region provides the regulation of sso II restriction-modification gene expression. Nucleic Acids Res..

[15] O'Driscoll J, Fitzgerald GF, van Sinderen D (2005). A dichotomous epigenetic mechanism governs expression of the LlaJI restriction/modification system. Mol. Microbiol..

[16] Som S, Friedman S (1994). Regulation of EcoRII methyltransferase: effect of mutations on gene expression and in vitro binding to the promoter region. Nucleic Acids Res..

[17] Protsenko A, Zakharova M, Nagornykh M, Solonin A, Severinov K (2009). Transcription regulation of restriction-modification system Ecl18kI. Nucleic Acids Res..

[18] Liu Y, Kobayashi I (2007). Negative regulation of the EcoRI restriction enzyme gene is associated with intragenic reverse promoters. J. Bacteriol..

[19] Mruk I, Liu Y, Ge L, Kobayashi I (2011). Antisense RNA associated with biological regulation of a restriction-modification system. Nucleic Acids Res..

[20] Nagornykh M, Zakharova M, Protsenko A, Bogdanova E, Solonin AS, Severinov K (2011). Regulation of gene expression in restriction-modification system Eco29kI. Nucleic Acids Res..

[21] Mruk I, Rajesh P, Blumenthal RM (2007). Regulatory circuit based on autogenous activation-repression: roles of C-boxes and spacer sequences in control of the PvuII restriction-modification system. Nucleic Acids Res..

[22] Sorokin V, Severinov K, Gelfand MS (2009). Systematic prediction of control proteins and their DNA binding sites. Nucleic Acids Res..

[23] Tao T, Bourne JC, Blumenthal RM (1991). A family of regulatory genes associated with type II restriction-modification systems. J. Bacteriol..

[24] Mruk I, Blumenthal RM (2008). Real-time kinetics of restriction-modification gene expression after entry into a new host cell. Nucleic Acids Res..

[25] Hermsen R, Erickson DW, Hwa T (2011). Speed, sensitivity, and bistability in auto-activating signaling circuits. PLoS Comput. Biol..

[26] Igoshin OA, Alves R, Savageau MA (2008). Hysteretic and graded responses in bacterial two-component signal transduction. Mol. Microbiol..

[27] Savageau MA (2002). Alternative designs for a genetic switch: analysis of switching times using the piecewise power-law representation. Math. Biosci..

[28] Vijesurier RM, Carlock L, Blumenthal RM, Dunbar JC (2000). Role and mechanism of action of C. PvuII, a regulatory protein conserved among restriction-modification systems. J. Bacteriol..

[29] Becskei A, Serrano L (2000). Engineering stability in gene networks by autoregulation. Nature.

[30] Savageau MA (1974). Comparison of classical and autogenous systems of regulation in inducible operons. Nature.

[31] Hu J, Qin KR, Xiang C, Lee TH (2012). Modeling of hysteresis in gene regulatory networks. Bull. Math. Biol..

[32] Fasani RA, Savageau MA (2010). Automated construction and analysis of the design space for biochemical systems. Bioinformatics.

[33] Schwartz R (2008). Biological Modeling and Simulation: A Survey of Practical Models, Algorithms, and Numerical Methods.

[34] Guzman LM, Belin D, Carson MJ, Beckwith J (1995). Tight regulation, modulation, and high-level expression by vectors containing the arabinose PBAD promoter. J. Bacteriol..

[35] Wild J, Hradecna Z, Szybalski W (2002). Conditionally amplifiable BACs: switching from single-copy to high-copy vectors and genomic clones. Genome Res..

[36] Knowle D, Lintner RE, Touma YM, Blumenthal RM (2005). Nature of the promoter activated by C.PvuII, an unusual regulatory protein conserved among restriction-modification systems. J. Bacteriol..

[37] Brosius J (1984). Plasmid vectors for the selection of promoters. Gene.

[38] Choi-Rhee E, Cronan JE (2003). The biotin carboxylase-biotin carboxyl carrier protein complex of Escherichia coli acetyl-CoA carboxylase. J. Biol. Chem..

[39] Bogdanova E, Djordjevic M, Papapanagiotou I, Heyduk T, Kneale G, Severinov K (2008). Transcription regulation of the type II restriction-modification system AhdI. Nucleic Acids Res..

[40] Savageau MA (2001). Design principles for elementary gene circuits: elements, methods, and examples. Chaos.

[41] Wall ME, Hlavacek WS, Savageau MA (2003). Design principles for regulator gene expression in a repressible gene circuit. J. Mol. Biol..

[42] Wall ME, Hlavacek WS, Savageau MA (2004). Design of gene circuits: lessons from bacteria. Nat. Rev. Genet..

[43] Igoshin OA, Price CW, Savageau MA (2006). Signalling network with a bistable hysteretic switch controls developmental activation of the sigma transcription factor in Bacillus subtilis. Mol. Microbiol..

[44] Wong Ng J, Chatenay D, Robert J, Poirier MG (2010). Plasmid copy number noise in monoclonal populations of bacteria. Phys. Rev. E Stat. Nonlin. Soft Matter Phys..

[45] Luo ZQ, Su S, Farrand SK (2003). In situ activation of the quorum-sensing transcription factor TraR by cognate and noncognate acyl-homoserine lactone ligands: kinetics and consequences. J. Bacteriol..

[46] Proshkin S, Rahmouni AR, Mironov A, Nudler E (2010). Cooperation between translating ribosomes and RNA polymerase in transcription elongation. Science.

[47] Dalbow DG, Young R (1975). Synthesis time of beta-galactosidase in Escherichia coli B/r as a function of growth rate. Biochem. J..

[48] Siegele DA, Hu JC (1997). Gene expression from plasmids containing the araBAD promoter at subsaturating inducer concentrations represents mixed populations. Proc. Natl Acad. Sci. USA.

[49] Khlebnikov A, Risa O, Skaug T, Carrier TA, Keasling JD (2000). Regulatable arabinose-inducible gene expression system with consistent control in all cells of a culture. J. Bacteriol..

[50] Hiszczynska-Sawicka E, Kur J (1997). Effect of Escherichia coli IHF mutations on plasmid p15A copy number. Plasmid.

[51] Beckett D, Kovaleva E, Schatz PJ (1999). A minimal peptide substrate in biotin holoenzyme synthetase-catalyzed biotinylation. Protein Sci..

[52] Pfeuty B, Kaneko K (2009). The combination of positive and negative feedback loops confers exquisite flexibility to biochemical switches. Phys. Biol..

[53] Mitrophanov AY, Groisman EA (2008). Positive feedback in cellular control systems. BioEssays: news and reviews in molecular, cellular and developmental biology.

[54] Atkinson MR, Savageau MA, Myers JT, Ninfa AJ (2003). Development of genetic circuitry exhibiting toggle switch or oscillatory behavior in Escherichia coli. Cell.

[55] Beckett D, Kovaleva E, Schatz PJ (1999). A minimal peptide substrate in biotin holoenzyme synthetase-catalyzed biotinylation. Protein Sci..

[56] Mruk I, Blumenthal RM (2009). Tuning the relative affinities for activating and repressing operators of a temporally regulated restriction-modification system. Nucleic Acids Res..

[57] McGeehan JE, Papapanagiotou I, Streeter SD, Kneale GG (2006). Cooperative binding of the C.AhdI controller protein to the C/R promoter and its role in endonuclease gene expression. J. Mol. Biol..

[58] Cooper S, Helmstetter CE (1968). Chromosome replication and the division cycle of Escherichia coli B/r. J. Mol. Biol..

[59] Cooper S (2006). Regulation of DNA synthesis in bacteria: analysis of the Bates/Kleckner licensing/initiation-mass model for cell cycle control. Mol. Microbiol..

[60] Blumenthal RM (1977). Physiology of transcription termination factor rho in Escherichia coli. Ph.D. Thesis.

[61] Klumpp S (2011). Growth-rate dependence reveals design principles of plasmid copy number control. PLoS One.

