# Supplementary material for: A bistable hysteretic switch in an activator–repressor regulated restriction–modification system
Source: Nucleic Acids Res. 2013 Apr 27;41(12):6045–57. doi: 10.1093/nar/gkt324 (PMC3695507; doi:10.1093/nar/gkt324)
Supplement: Supplementary Data [file supp_41_12_6045__index.html]

A bistable hysteretic switch in an activator–repressor regulated restriction–modification system — A bistable hysteretic switch in an activator–repressor regulated restriction–modification system — Supplementary Data 

# A bistable hysteretic switch in an activator–repressor regulated restriction–modification system

## Supplementary Data

files

**Files in this Data Supplement:**

- Supplementary Data - pdf file
