# Supplementary material for: A bistable hysteretic switch in an activator–repressor regulated restriction–modification system
Source: Nucleic Acids Res. 2013 Apr 27;41(12):6045–57. doi: 10.1093/nar/gkt324 (PMC3695507; doi:10.1093/nar/gkt324)
Supplement: Supplementary Data [file supp_gkt324_nar-00640-h-2013-File010.pdf]

SUPPLEMENTARY INFORMATION FOR:

A bistable hysteretic switch in an activator-repressor regulated restriction-modification system

Kristen Williams,[1] Michael A. Savageau,[2] and Robert M. Blumenthal[1]*

[1] Department of Medical Microbiology & Immunology, and Program in Bioinformatics, University of Toledo, Toledo, OH 43614, USA; and [2] Biomedical Engineering Department, and Microbiology Graduate Group, University of California, Davis, CA 95616, USA

* To whom correspondence should be addressed. Tel: +01 419 383 5422; Fax: +01 419 383 3002; Email: Robert.Blumenthal@utoledo.edu

Table S1. Oligonucleotide primer pairs used for QRT PCR

Target	Primer sequence
pvuIIM	5'- AAACGCCGATGCCGCAACATATTC
	5'- TTGATGGGTATTAAAGCGCATCCCG
pvuIIR	5'- TGGTGGAAAGTTGCTTCAAGTCCT
	5'- TGCGATACCACGGTATATGGCAAA
pvuIIC	5'- CAAATCCTTTATCAGCCCGATTAACCC
	5'- AGGCATTTGCTATTCGCTCAATGT
cat	5'- ATCACAAACGGCATGATGAA
	5'- GCGTGTTACGGTGAAAACCT
amp	5'- ATAATACCGGCGCACATAGC
	5'- TTTGCCTTCCTGTTTTTGCT
lacZ	5'- ACTATCCCGACCGCCTTACT
	5'- TAGCGGCTGATGTTGAACTG
kan	5'- TTATGCCTCTTCCGACCATC
	5'- GCCTGAGCGAGACGAAATAC
tet	5'- GACAGCATCGCCAGTCACTA
	5'- GCGTAGAGGATCCACAGGAC

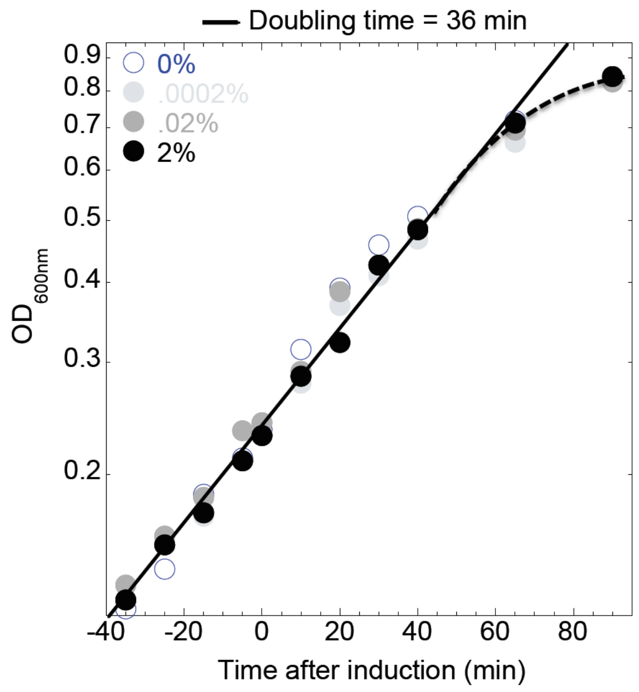

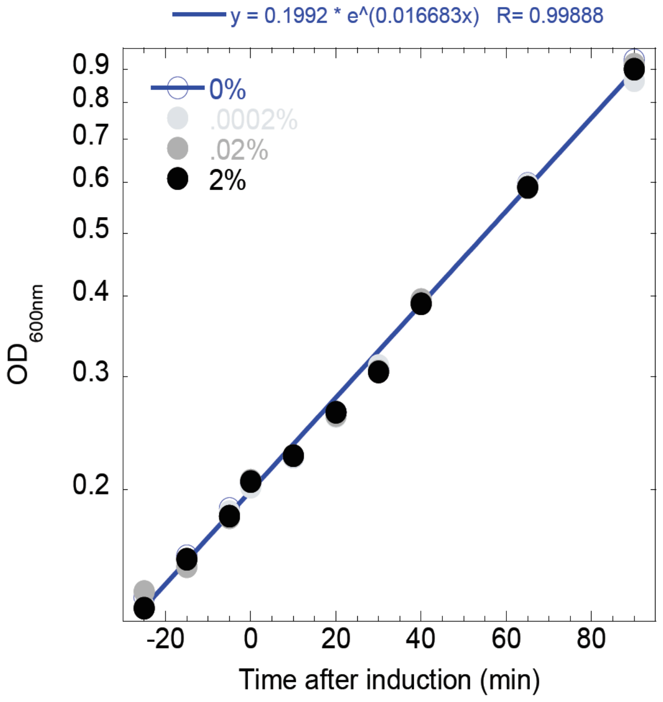

Figure S1. Growth curves for induction kinetics cultures. Two strains are being used, both in the *E. coli* TOP10 background, that differ only in the nature of the plasmid-borne P*pvuIICR* promoter: either WT or a nonrepressing mutant. These cultures were used for the experiments shown in Figure 4. **A.** WT promoter/operator strain. At time = 0, the indicated amount of arabinose was added to a culture growing logarithmically in MOPS-rich medium. No significant effect was seen on growth; doubling time was ~36 min. **B.** Nonrepressing promoter strain. The cultures gave a doubling time of 41.6 min (~15% slower than WT).

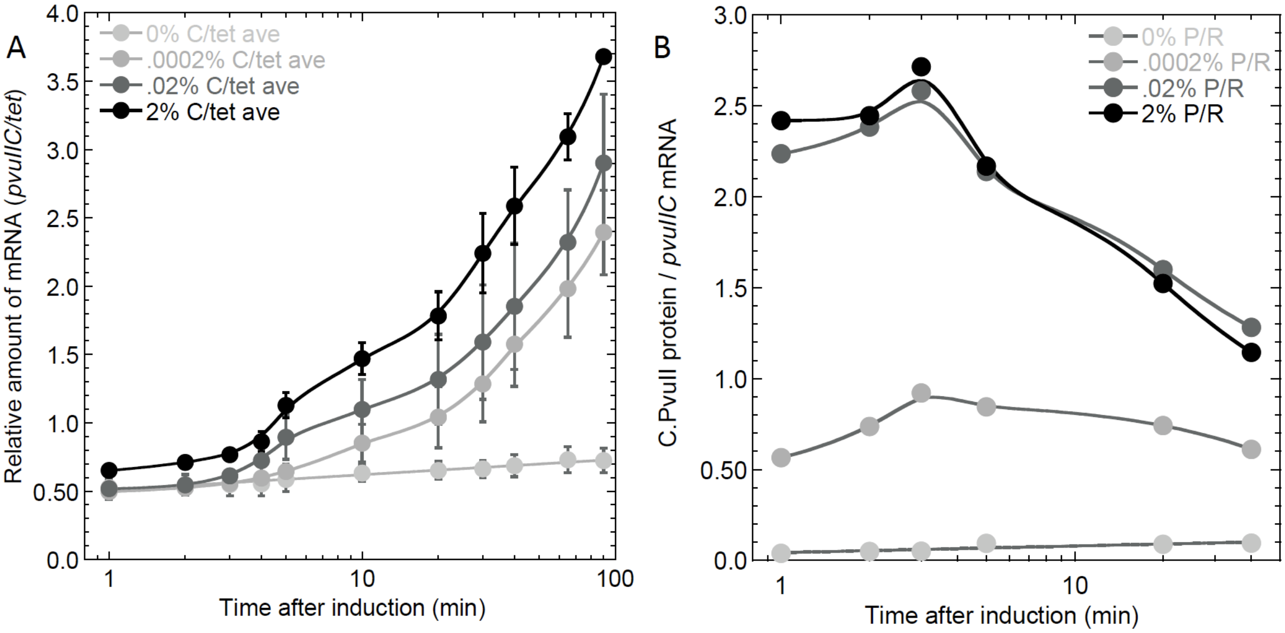

Figure S2. Induction profile of *pvuIIC*. A. *pvuIIC* mRNA divided by *tet* mRNA. At time = 0, various amounts of arabinose were added to an exponentially-growing culture in MOPS rich medium, and QRT-PCR was used to measure mRNA levels. Data shown are the averages (± range) from two independent experiments using the two *E. coli* TOP10 strains used in this work. One is the strain carrying the WT promoter/operator (on a second plasmid), while the second is the strain with nonrepressing operator on the second plasmid. In both cases, the strain background and the plasmid carrying the P*araBAD-pvuIIC* fusion are identical. **B.** Protein/mRNA ratios. The protein levels are from Figure 3B, and the mRNA levels are from (A).

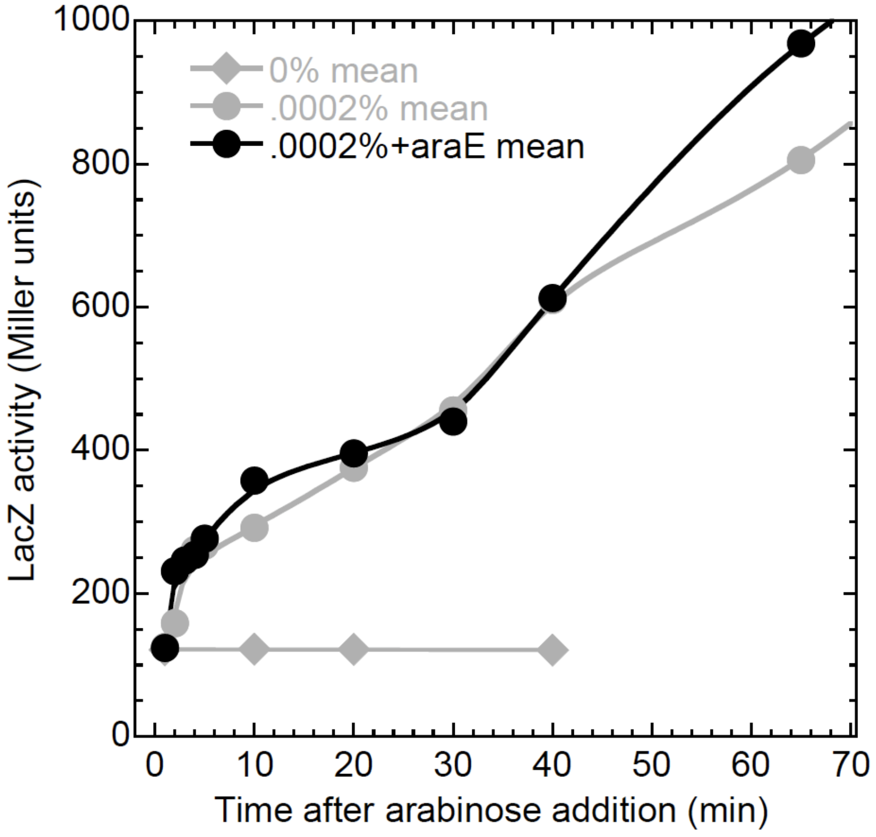

Figure S3. Effect of constitutive *araE* expression on arabinose induction kinetics. At lower concentrations of the inducer arabinose, induction of the arabinose transporter gene *araE* might make a subpopulation of cells more responsive to the inducer. Strains carrying the plasmids shown in Figure 4, with (black circles) or without (gray circles) the cloned *araE* gene added to the plasmid carrying the *pvuIIC* gene, were grown and assayed as in Figure 5, panel C. The SE bars from triplicates are plotted but smaller than the symbols.
